# Do disordered eating behaviours in girls vary by school characteristics? A UK cohort study

**DOI:** 10.1007/s00787-018-1133-0

**Published:** 2018-03-15

**Authors:** Helen Bould, Bianca De Stavola, Glyn Lewis, Nadia Micali

**Affiliations:** 1 Department of Psychiatry, University of Oxford, Warneford Lane, Oxford, UK; 2 GOS Institute of Child Health, UCL, London, UK; 3 Division of Psychiatry, UCL, London, UK; 4 Department of Psychiatry, University of Geneva, Geneva, Switzerland; 5 Child and Adolescent Psychiatry Division, Department de l’Enfant et de l’Adolescent, HUG, Geneva, Switzerland

**Keywords:** Eating disorders, Disordered eating, Body dissatisfaction, ALSPAC

## Abstract

Previous research on eating disorders, disordered eating behaviours, and whether their prevalence varies across schools, has produced inconsistent results. Our previous work using Swedish record-linkage data found that rates of diagnosed eating disorders vary between schools, with higher proportions of girls and higher proportions of highly educated parents within a school being associated with greater numbers of diagnosed eating disorders. We aimed to extend these findings to a UK population-based sample and hypothesised that a similar association would be evident when studying disordered eating behaviours. We used data from the Avon Longitudinal Study of Parents and Children to test the hypothesis that prevalence of self- and parent-reported disordered eating behaviours (binge eating, purging, fasting, restrictive eating, and fear of weight gain), and body dissatisfaction cluster by school. We had complete data on body dissatisfaction, school attended, and other possible risk factors for 2146 girls in 263 schools at age 14 and on disordered eating behaviours for 1769 girls in 273 schools at age 16. We used multilevel logistic regression modelling to assess whether prevalence varied between and within schools, and logistic regression to investigate the association between specific school characteristics and prevalence of disordered eating behaviours and body dissatisfaction. At age 14, there was no evidence for body dissatisfaction clustering by school, or for specific school characteristics being associated with body dissatisfaction. At age 16, there was no evidence for clustering, but higher rates of disordered eating behaviours were associated with attending all-girl schools and lower levels with attending schools with higher academic results. We found no evidence for clustering of disordered eating behaviours in individual schools, possibly because of the small cluster sizes. However, we found evidence for higher levels of disordered eating behaviours in 16 years in all-girl schools, and in schools with lower academic performance.

**Electronic supplementary material:**

The online version of this article (10.1007/s00787-018-1133-0) contains supplementary material, which is available to authorized users.

## Introduction

Understanding the aetiology of eating disorders is important given their high prevalence [1–3], prospective associations with adverse outcomes [1, 4], high mortality [5], and healthcare costs. One possible modifiable risk factor is the school environment, since the peak of onset for eating disorders is during adolescence [6, 7]. It is, therefore, important to understand whether and how schools might be associated with risk of developing an eating disorder. Our recent record-linkage-based research [8] of Swedish girls who left high school between 2002 and 2010 examined differences in rates of diagnosed eating disorders between schools and found support for the clinical impression that diagnosed eating disorders are more common in some schools than others. The study found that rates of diagnosed eating disorders were higher in schools with greater proportions of female students and in schools with greater proportions of highly educated parents. Differences between schools withstood adjustment for individual student characteristics, supporting the hypothesis that the school environment could influence eating disorder incidence. However, the outcome was based on subjects who accessed secondary care services and who were given a diagnosis of an eating disorder. Since less than 30% of individuals with ED report seeking help for their eating disorder in the UK [9], and only 3–28% of those with a diagnosed eating disorder report receiving specific treatment for weight and eating problems in the US [10], it is important to study these symptoms at a population level. Population studies in the US have found that disordered weight control behaviours also vary between schools, but that differences do not persist following adjustment for individual risk factors [11], and that the likelihood that an individual female student is trying to lose weight increases with the proportion of underweight girls in her school [12].

Schools have been found to play a role in other adolescent health problems. For example, the prevalence of tobacco, alcohol, and illicit substance use varies between schools [13], and aggregate pupil disapproval of substance use is associated with lower substance use, after adjusting for individual views [14]. Lower school average income is independently associated with higher rates of depression in students, after accounting for individual factors including household income [15]; higher proportions of students of lower socioeconomic status and lower school academic performance are associated with increased rates of self-harm [16]. Schools constitute a potential route for intervention to prevent eating disorders and ensure their early recognition and treatment, as recommended by the UK Chief Medical Officer order to improve recovery rates [17].

The Avon Longitudinal Study of Parents and Children (ALSPAC) allows ascertainment of disordered eating behaviours in the community, rather than only in those who access treatment and receive a diagnosis. This study first investigated whether body dissatisfaction at age 14 and disordered eating behaviours (binging, purging and fasting, restrictive eating, and fear of weight gain) at age 16 cluster in particular schools, with no a priori hypothesis about association of body dissatisfaction or disordered eating behaviours with specific school characteristics. Secondly, we hypothesised that specific school characteristics, such as all girls versus mixed schools, schools with higher versus lower academic attainment, schools with less versus more pastoral care, and schools with higher versus lower than average levels of deprivation would be associated with greater levels of body dissatisfaction and disordered eating behaviours.

## Methods

### Sample

The sample comprised participants from the Avon Longitudinal Study of Parents and Children (ALSPAC: http://www.alspac.bris.ac.uk). ALSPAC is an ongoing population-based study investigating a wide range of influences on health and development of children. Ethical approval for the study was obtained from the ALSPAC Law and Ethics Committee and the Local Research Ethics Committees. Pregnant women resident in the former Avon Health Authority in south-west England, having an estimated date of delivery between 1/4/91 and 31/12/92 were invited to take part, resulting in a cohort of 14,541 pregnancies and 13,988 children (*n* = 6762 girls) alive at 12 months of age. When the oldest children were aged 7 years, an attempt was made to increase the size of the initial sample with eligible cases that did not join the cohort at the outset. The number of active new cases that are represented in the data resource is 713 (*n* = 392 girls). The phases of enrolment are described in more detail in the cohort profile paper [18]. Amongst twin pairs, one twin per pair was randomly excluded. Please note that the study website contains details of all the data that is available through a fully searchable data dictionary (http://www.bris.ac.uk/alspac/researchers/data-access/data-dictionary).

At age 14, we had data on school attended by 5031 female participants. Of these, 2627 were missing data on body satisfaction or weight and shape concern, and 258 were missing data for other possible explanatory variables, leaving 2146 participants. At age 16, we had data on school attended for 5260 female participants. Of these, 2991 were missing data on disordered eating behaviours, and a further 500 were missing data on other possible explanatory variables, leaving 1769 participants. Figures 1a and 1b (online supplements) show flow charts with further details of how the samples with complete data were derived.

### Measures

#### Weight and shape concern and body dissatisfaction

Weight and shape concern were measured at age 14 using 3 questions from the McKnight Risk Factor Survey [19] to derive a dichotomous variable, as used previously with ALSPAC data [20]. Body dissatisfaction was assessed using the Body Dissatisfaction Scale [21], which asks participants to rate their satisfaction with nine body parts on a four-point Likert Scale from ‘extremely satisfied’ to ‘extremely dissatisfied’, creating a continuous scale.

#### Disordered eating behaviours

Data on disordered eating behaviours (binge eating, purging, and fasting) were gathered at ages 14 and 16, using questions adapted from the Youth Risk Behaviour Surveillance System questionnaire [22], enquiring about the previous year. We chose to investigate it at age 16, as the prevalence of individual behaviours was low at age 14. Binge eating was assessed as present in adolescents who reported eating a very large amount of food at least once a week, and feeling out of control during these episodes. Purging was assessed by asking how many times in the past year the adolescent had made themselves sick or used laxatives to lose weight or prevent weight gain. These questions have been validated in comparison with the Eating Behaviours Interview in a population-based sample of adolescents [23], where the purging questions had a sensitivity of 0.93 and a specificity of 0.86, and the binge eating questions had a sensitivity of 0.53 and a specificity of 0.79. Fasting was assessed with the question “During the past year, how often did you fast (not eat for at least half a day) to lose weight or avoid gaining weight?” Binge eating, fasting, and purging were all used as dichotomous variables (ever or never engaged in this behaviour). An additional composite dichotomous measure indicating the presence of any disordered eating behaviour was generated from these data, and a further dichotomous measure was created for where frequency of these behaviours met DSM 5 diagnostic criteria.

Parental questionnaires based on the Development and Wellbeing Assessment (DAWBA) [24] were also completed at ages 14 and 16. We used the questions concerning restrictive eating and fear of weight gain, as these are key symptoms of anorexia nervosa. Each question could be answered “no”, “a little”, or “a lot”. We created a composite dichotomous variable from these questions, where parental report of “a lot” on either or both questions resulted in a score of 1, and other responses in a score of 0.

#### Explanatory individual-level factors

Analyses included a range of potential individual-level explanatory factors derived from self-report postal questionnaires. Maternal age at birth of the study participant was included. Maternal educational level has been found to be associated with eating disorders in offspring [25, 26] and was, therefore, included as an ordinal variable [(1) advanced-level qualifications generally obtained at age 18 years and required for university entry)/university degree; (2) ordinary-level qualifications generally obtained at age 16 years; (3) certificate of secondary school education (lowest level qualifications generally obtained at age 16 years)/vocational/none]. The highest social class of mother or father during pregnancy [I (professional), II (managerial/technical), IIIN (skilled non-manual), IIIM (skilled manual), IV (partly skilled), V (unskilled)] was included as an ordinal variable, as high SES has been found to be associated with anorexia nervosa in offspring [27], and in contrast, family economic disadvantage is associated with body dissatisfaction and binge eating [20]. Parity was included as an ordinal variable coded from 0 to 3, with 3 including three or more children, as there is some conflicting evidence from case–control studies as to whether birth order may alter risk of eating disorders [28–30]. Maternal self-reported history of an eating disorder (during pregnancy, or when the study child was aged 7) was included as a binary variable, given the association between maternal eating disorders and both eating disorders [31] and body dissatisfaction [20] in children. Finally, maternal depression at 32 weeks of pregnancy (Edinburgh Postnatal Depression Scale) [32] was included as a binary variable, using a score greater than 12 to define a case of depression, as maternal depression has been found to be associated with eating disorders in offspring [33].

#### Explanatory school-level factors

Information on which schools were attended by the subjects is available for Key Stage 3 (students aged 14) and Key Stage 4 (students aged 16). This meant that we had access to data on a range of school-level variables, which we were able to use to investigate school characteristics associated with a higher risk of disordered eating behaviours. We used the following potentially explanatory binary school-level variables: all girl versus mixed schools, average school exam grade (Key Stage 3 or 4) in the top 25% versus the bottom 75% of schools, the presence or absence of a school nurse, and having fewer or greater than 5% of children at the school in receipt of free school meals.

### Statistical analysis

We described the whole sample and compared it with the sample with complete data for both ages and for all covariates (“Complete Case Sample”) (Table 1). We described differences in prevalence of body dissatisfaction and disordered eating behaviours (both self- and parental reports) as recorded at age 14 and 16 years by individual and school characteristics (Table 2). We then assessed whether there was statistical evidence of clustering in body dissatisfaction and disordered eating behaviours among pupils from the same school by fitting mixed effects linear (for body satisfaction) and logistic (for weight and shape concern, and all the disordered eating behaviours) regression models [34]. This allowed us to calculate and then compare the intraclass correlation coefficient (ICC) corresponding to each fitted model. This analysis ignores any information we have about individual schools and looks for clustering irrespective of any hypotheses we might have about particular school characteristics.

**Table 1:** Comparison of whole sample with complete case sample by age

	Age 14 years		Age 16 years	
	Whole sample (*N* varies by covariate)	Complete case sample (*N* = 2146)	Whole sample (*N* varies by covariate)	Complete case sample (*N* = 1769)
Number of ALSPAC girls per school (median, 25th centile, 75th centile)	1 (1, 2)	1 (1,2)	1 (1,2)	1 (1,2)
Body Dissatisfaction score (mean, SD)	23.8 (8.05) (*N* = 2404)	23.8 (8.0)	–	–
Weight and shape concern [*N* (%) scoring > 7]	451/2404 (18.8)	383 (17.9)	–	–
Purging [*N* (%)]	–	–	223/2346 (9.5)	167 (9.4)
Binge eating [*N* (%)]	–	–	384/2346 (16.4)	278 (15.7)
Fasting [*N* (%)]	–	–	481/2346 (20.5)	360 (20.4)
Any compensatory behaviour [*N* (%)]	–	–	972/2346 (41.4)	718 (40.6)
Any compensatory behaviour (DSM 5 frequency) [*N* (%)]	–	–	285/2346 (12.2)	202 (11.4)
Parent-reported fat avoidance/fear of weight gain (*N*, % scoring ≥ 3)	232/1940 (12.0)	205/1770 (11.6)	242/1778 (13.6)	183/1441 (12.7)
First born child (*N*, %)	1077/2331 (46.2)	996 (46.4)	1074/2274 (47.2)	841 (47.5)
Mother’s age at child’s birth (mean, SD)	28.6 (4.6) (*N* = 2404)	28.7 (4.4)	29.0 (4.6) (*N* = 2346)	29.2 (4.4)
Maternal education of A level or above (*N*, %)	908/2327 (39)	858 (40.0)	979/2275 (43.0)	801 (45.3)
Parental occupational class [class I (*N*, %]	288/2216 (13.0)	278 (13.0)	332/2176 (15.3)	289 (16.3)
Maternal self-report history of ED [*N*, %]	110/2404 (4.6)	98 (4.6)	86/1995 (4.3)	71 (4.0)
Maternal antenatal depression (EPDS)	298/2265 (13.2)	271 (12.6)	262/2212 (11.8)	200 (11.3)
Average KS3 point score of their school (mean, SD)	37.9 (5.8) (*N* = 1984)	38.2 (5.6) (*N* = 1790)	–	–
Average KS4 point score of their school (mean, SD)	–	–	470 (130) (*N* = 2345)	485 (122) (*N* = 1768)
Number of students at their school (mean, SD)	1224 (317) (*N* = 2404)	1234 (316)	1180 (363) (*N* = 2270)	1195 (362)
Proportion of girls in their school (mean, SD)	0.51 (0.10) (*N* = 2404)	0.52 (0.10)	0.53 (0.15) (2269)	0.53 (0.15)
Attend an all-girl school (*N*, %)	91/2404 (3.8)	82 (3.8)	187/2269 (8.2)	147 (8.3)
Have healthcare staff in their school (*N*, %)	945/2404 (39.3)	867 (40.0)	792/2270 (34.9)	631 (35.7)
At a school where ≥ 5% students have free school meals	1039/2404 (43.2)	892 (41.6)	788/2270 (34.7)	592 (33.5)

**Table 2:** Comparison of means and percentages of disordered eating behaviours between schools with different characteristics

	Age 14			Age 16					
	Body Dissatisfaction score (mean, 95% confidence intervals) (*N* = 2146)	Weight/shape concern above cut off (7) (%, 95% confidence intervals) (*N* = 2146)	Parental report of fear of/avoidance of fat (% score ≥ 3; 95% Confidence Intervals) (*N* = 1770)	Fasting (%, 95% confidence intervals) (*N* = 1769)	Purging (%, 95% confidence intervals) (*N* = 1769)	Binge eating (%, 95% confidence intervals) (*N* = 1769)	Any compensatory behaviour (%, 95% confidence intervals) (*N* = 1769)	Any compensatory behaviour (DSM-5 intensity) (%, 95% confidence intervals) (*N* = 1769)	Parental report of fear of/avoidance of fat (score ≥ 3) (%, confidence intervals) (*N* = 1441)
Girls at all-girl schools	22.9 (21.4, 24.4) (*N* = 82)	13.4 (5.8, 20.9) (*N* = 82)	18.1 (9.0, 27.2) (*N* = 72)	21.1 (14.4, 27.8) (*N* = 147)	11.6 (6.3, 16.8) (*N* = 147)	17.0 (10.9, 23.2) (*N* = 147)	48.3 (40.1, 56.5) (*N* = 147)	12.9 (7.4, 18.4) (*N* = 147)	14.0 (7.9, 20.0) (*N* = 129)
Girls at mixed schools	23.8 (23.5, 24.2) (*N* = 2313)	18.0 (16.4, 19.7) (*N* = 2064)	11.3 (9.8, 12.8) (*N* = 1698)	20.3 (18.3, 22.2) (*N* = 1622)	9.2 (7.8, 10.7) (*N* = 1622)	15.6 (13.8, 17.4) (*N* = 1622)	39.9 (37.5, 42.3) (*N* = 1622)	11.3 (9.7, 12.8) (*N* = 1622)	12.6 (10.8, 14.4) (*N* = 1312)
Girls at schools in top 25% KS3/KS4 point score total	23.1 (22.4, 23.8) (*N* = 473)	14.2 (11.0, 17.3) (*N* = 473)	9.5 (6.7, 12.3) (*N* = 421)	14.8 (11.4, 18.1) (*N* = 440)	8.0 (5.5, 10.5) (*N* = 440)	15.2 (11.9, 18.6), *N* = 440)	38.6 (34.1, 43.2) (*N* = 440)	7.5 (5.0, 10.0) (*N* = 440)	11.2 (8.0, 14.4) (*N* = 376)
Girls at schools not in top 25% of KS3/KS4 point score total	24.1 (23.7, 24.6) (*N* = 1317) (Total *N* = 1790)	19.5 (17.4, 21.7) (*N* = 1317) (Total *N* = 1790)	12.2 (10.2, 14.2) (*N* = 1056)	22.2 (20.0, 24.5) (*N* = 1328) (Total *N* = 1768)	9.9 (8.3, 11.6) (*N* = 1328) (Total *N* = 1768)	15.8 (13.8, 17.8) (*N* = 1328) (Total *N* = 1768)	41.3 (38.6, 43.9) (*N* = 1328) (Total *N* = 1768)	12.7 (10.9, 14.5) (*N* = 1328) (Total *N* = 1768)	13.3 (11.2, 15.3) (*N* = 1064) (Total *N* = 1440)
Girls at schools with school nurse	23.4 (22.9, 24.0) (*N* = 867)	15.1 (12.7, 17.5) (*N* = 867)	10.4 (8.1, 12.6) (*N* = 723)	19.5 (16.4, 22.6) (*N* = 631)	9.8 (7.5, 12.2), (*N* = 631)	15.1 (12.3, 17.9) (*N* = 631)	42.2 (38.3, 46.0) (*N* = 631)	11.3 (8.8, 13.7) (*N* = 631)	11.7 (8.9, 14.5) (*N* = 513)
Girls at schools with no school nurse	24.0 (23.6, 24.5) (*N* = 1279)	19.7 (17.5, 21.9) (*N* = 1279)	12.4 (10.4, 14.4) (*N* = 1047)	20.8 (18.5, 23.2) (*N* = 1138)	9.2 (7.5, 10.9) (*N* = 1138)	16.1 (13.9, 18.2) (*N* = 1138)	39.7 (36.9, 42.6) (*N* = 1138)	11.5 (9.7, 13.4) (*N* = 1138)	13.3 (11.1, 15.4) (*N* = 928)
Girls at schools with ≤ 5% of students entitled to free school meals	23.7 (23.2, 24.1) (*N* = 1254)	17.0 (14.9, 19.1) (*N* = 1254)	11.1 (9.2, 13.0) (*N* = 1045)	20.4 (18.1, 22.7) (*N* = 1177)	9.8 (8.1, 11.5) (*N* = 1177)	15.6 (13.6, 17.7) (*N* = 1177)	41.2 (38.4, 44.0) (*N* = 1177)	11.5 (9.6, 13.3) (*N* = 1177)	12.3 (10.2, 14.3) (*N* = 978)
Girls at schools with > 5% on free school meals	23.9 (23.4, 24.5) (*N* = 892)	19.1 (16.5, 21.6) (*N* = 892)	12.3 (9.9, 14.7) (*N* = 725)	20.3 (17.0, 23.5) (*N* = 592)	8.8 (6.5, 11.1) (*N* = 592)	15.9 (12.9, 18.8) (*N* = 592)	39.4 (35.4, 43.3) (*N* = 592)	11.3 (8.8, 13.9) (*N* = 592)	13.6 (10.5, 16.7) (*N* = 463)

We then calculated mean difference in body dissatisfaction and odds ratios for the risk of experiencing disordered eating behaviours according to school characteristics (all girl versus mixed; school in the top 25% versus bottom 75% exam scores compared with other schools; the presence or absence of school health nurse; ≤ 5 versus > 5% of the students being eligible for free school meals) (Table 3). These analyses were repeated adjusting for individual characteristics, other school characteristics, and both together.

**Table 3:** Comparison of disordered eating behaviours between schools with different characteristics [expressed as odds ratios (ORs) or regression coefficients]; adjusted for school and individual characteristics

	Age 14			Age 16					
	Body Dissatisfaction score (regression coeff, 95% Confidence Interval) (*N* = 1790)	Weight/shape concern above cut off (7) (OR, 95% confidence interval) (*N* = 1790)	Parental report of fear of/avoidance of fat (score ≥ 3) (OR, 95% confidence interval) (*N* = 1477)	Fasting (OR, 95% confidence interval) (*N* = 1759)	Purging (OR, 95% confidence interval) (*N* = 1759)	Binge eating (OR, 95% confidence interval) (*N* = 1768)	Any compensatory behaviour (OR, 95% confidence interval) (*N* = 1768)	Any compensatory behaviour (DSM-5 intensity) (OR, 95% confidence interval) (*N* = 1768)	Parental report of fear of/avoidance of fat (score ≥ 3) (OR, confidence interval) (*N* = 1436)
All girls versus mixed schools	− 0.06 (− 0.16, 0.04), *p* = 0.26	0.62 (0.27, 1.39), *p* = 0.24	1.88 (0.90, 4.02),* p* = 0.08	1.14 (0.74, 1.76), *p* = 0.55	1.43 (0.81, 2.51), *p* = 0.22	1.10 (0.69, 1.77), *p* = 0.68	1.48 (1.04, 2.10),* p* = 0.03	1.30 (0.76, 2.22), *p* = 0.33	1.11 (0.64, 1.93), *p* = 0.71
Top 25% versus bottom 75% KS point score total	− 0.02 (− 0.06, 0.02), *p* = 0.27	0.83 (0.60, 1.14), *p* = 0.24	0.88 (0.59, 1.32), *p* = 0.54	0.61 (0.45, 0.83),* p* = 0.001	0.76 (0.51, 1.13), *p* = 0.18	0.95 (0.70, 1.30), *p* = 0.76	0.88 (0.70, 1.10), *p* = 0.26	0.59 (0.40, 0.89),* p* = 0.01	0.82 (0.56, 1.19), *p* = 0.30
School nurse versus no school nurse	− 0.03 (− 0.06, 0.01), *p* = 0.15	0.83 (0.63, 1.08), *p* = 0.16	0.86 (0.60, 1.22), *p* = 0.39	0.93 (0.72, 1.19), *p* = 0.55	1.08 (0.77, 1.53), *p* = 0.65	0.94 (0.71, 1.24), *p* = 0.66	1.12 (0.92, 1.38), *p* = 0.26	0.98 (0.72, 1.35), *p* = 0.93	0.90 (0.64, 1.26), *p* = 0.54
≤ 5% versus > 5% on free school meals	− 0.003 (− 0.04, 0.04), *p* = 0.89	0.97 (0.75, 1.26), *p* = 0.84	1.00 (0.71, 1.41), *p* = 0.99	0.97 (0.75, 1.26), *p* = 0.82	0.95 (0.65, 1.37), *p* = 0.77	1.00 (0.75, 1.34), *p* = 0.99	0.98 (0.79, 1.22), *p* = 0.89	0.91 (0.65, 1.28), *p* = 0.59	1.12 (0.79, 1.60), *p* = 0.52

Birth order, maternal age, maternal education, parental social class, maternal depression, maternal eating disorder, all girls versus mixed school, top 25% versus bottom 75% KS point score total, school nurse; ≤ 5 versus > 5% on free school meal

## Results

### Descriptive data

The complete case sample consisted of 2146 girls from 254 schools at age 14, and 1769 girls from 273 schools at age 16. Schools had between 1 and 98 students who were part of ALSPAC at age 14 (median 1, 25th centile 1; 75th centile 2) and between 1 and 78 students at age 16 (median 1, 25th centile 1; 75th centile 2). Individuals in the complete case sample had slightly lower rates of disordered eating behaviours compared to the overall sample. They also had marginally older, more educated mothers, with slightly lower rates of depression. Individuals in the complete case samples were more likely to attend a school with higher exam scores and slightly less likely to attend a school where over 5% of the students are eligible for free school meals (Table 1). In the 14-year-old sample, 17.9% had significant concerns about their shape and weight, and in the 16-year-old sample, 40.7% had some form of disordered eating behaviour (fasting, purging, or binge eating), 11.3% at a level compatible with a DSM-5 eating disorder diagnosis.

### Clustering by school

As described, we used multilevel modelling to test for clustering of disordered eating behaviours within schools. We found no evidence that disordered eating behaviours or body dissatisfaction at either age 14 or age 16 clustered by school (the estimated ICC was 0 for all models fitted). Results were unchanged by adding individual-level explanatory variables to the model. We therefore used the standard regression models to estimate the associations reported below.

#### Association between disordered eating behaviours and school characteristics

##### Age 14

At age 14, girls in schools with Key Stage 3 results in the top quartile were less likely to have high levels of dissatisfaction with their shape and weight [OR 0.68 (95% CI 0.51, 0.91), *p* = 0.01, online supplementary Table 1]; the presence of a school health nurse also appeared to be protective against high levels of dissatisfaction with weight and shape (OR 0.73 (95% CI 0.58, 0.91), *p* = 0.007, online supplement Table 1). However, neither of these results withstood adjustment for individual and other school characteristics (Table 3 and online supplementary Tables 2 and 3). There was also a suggestion that girls at all-girl schools were more likely to avoid, and have a fear of, fat, although confidence intervals included the possibility of no difference [adjusted OR 95% CI 1.88 (0.90, 4.02), *p* = 0.08].

##### Age 16

At age 16, girls at schools with higher Key Stage 4 results were less likely to report fasting (OR 0.61 (95% CI 0.45, 0.81), *p* = 0.001, online supplementary Table 1), or any compensatory behaviour at DSM 5 levels [OR 0.55 (95% CI 0.38, 0.82), *p* = 0.003, online supplementary Table 1]. These results withstood adjustment for both individual and other school characteristics (Table 3) [adjusted OR for fasting: 0.61 (95% CI 0.45, 0.83), *p* = 0.001; adjusted OR for any compensatory behaviour 0.59 (95% CI 0.40, 0.89), *p* = 0.01].

At age 16, girls attending all female schools had higher levels of compensatory behaviours than their co-educated peers [OR 1.41 (95% 1.00, 1.97), *p* = 0.05, online supplementary Table 1], a relationship which was strengthened by adjusting for individual and other school characteristics which may have confounded this relationship [OR 1.48 (95% CI 1.04, 2.10), *p* = 0.03, Table 3].

## Discussion

### Main findings

At age 16, girls in schools with higher levels of academic achievement were less likely to report any disordered eating behaviour (fasting, binge eating or purging), whilst girls at all female schools had higher levels of disordered eating behaviours than their co-educated peers. When we used multilevel modelling, we did not find any evidence to suggest that disordered eating behaviours or body dissatisfaction clustered in schools when we did not examine our specific hypotheses. However, it must be noted that, rather surprisingly, in this study we lacked sufficient numbers of students at individual schools to separate the within and between schools components of variance. We did find evidence that low achieving schools and all female schools are associated with an increased prevalence of disordered eating behaviour.

### Strengths and limitations

This study extends the previous work on diagnosed eating disorders [8] by investigating disordered eating behaviours in a large sample. Its strengths are the large sample size, and detailed questionnaire and measurement data on individuals. Presentation to clinical services was not necessary to gather data, and we had prospective information on potentially explanatory variables. Although this is a sizeable cohort, each of the individual schools studied had a median of 1 (at ages 14 and 16) student who was part of the ALSPAC cohort. It is, therefore, likely that we had insufficient information on the variation in rates of disordered eating behaviours between individual schools. Furthermore, cohort studies are affected by differential drop out, and this may have led to the sample being too homogenous to detect differences between schools. The use of self-report questionnaires rather than clinical interviews to determine the presence or the absence of symptoms means that there is a possibility that symptoms may have been either over or under-reported, as on one hand subjects may feel more comfortable disclosing sensitive information in a questionnaire, but on the other, they would not have the opportunity to explore what was meant by the questions.

### Comparison with previous findings

These results are consistent with our previous study using Swedish record-linkage data [8]; in that we found that girls at schools with a greater proportion of girls (in this case, at all-girl schools) have higher rates of self-reported disordered eating behaviours, as well as diagnosed eating disorders. However, in contrast to that study, where schools with more highly educated parents had greater numbers of diagnosed eating disorders, we found here that more academic school environments, in terms of exam results, may be protective against disordered eating behaviours. This may be a reflection of differential cultural expectations, or, perhaps more likely, a consequence of misclassification error, with girls in schools with more educated parents being more likely to access support and treatment when suffering from disordered eating.

In terms of clustering within individual schools, we did not replicate results from a US population study [11], which found initial differences in disordered weight control behaviours between schools (although these did not persist following adjustment for individual risk factors), or those in our record-linkage study [8], in which we found clear differences between schools in rates of diagnosed eating disorders.

There are several possible explanations for findings differing according to the use of population as opposed to record-linkage data. The reversal of the trend in relation with whether more academic schools are a risk or protective factor for disordered eating could possibly be explained if the clustering found in the Swedish record-linkage study was due to schools with greater proportions of highly educated parents being more adept at detecting students with difficulties and ensuring they were referred for treatment. Alternatively, it may be due to sociodemographic variables operating in different directions for disordered eating behaviours in the community in comparison with those presenting for treatment. This has been seen at an individual level, where higher socioeconomic status and maternal education are risk factors for diagnosed eating disorders [25, 26], but financial difficulties appear to be a risk factor for disordered eating behaviours seen in the community [20]. Another possible explanation is the difference between the Swedish and UK school system: Swedish students choose which “Gymnasium” (High School) to move to at 15, and Gymnasiums vary in terms of the programmes of study offered. This may mean that Gymnasiums have student bodies which are more different from each other than UK secondary schools. For example, some Gymnasiums may have large numbers of high achieving, perfectionistic girls, who are at greater risk of developing eating disorders.

The null finding with regard to clustering seems most likely to be due to the small numbers of ALSPAC students at individual schools. A further possibility is that rates of Anorexia Nervosa (the disorder predominantly seen in child and adolescent psychiatric services) do vary between schools, but we do not have sufficient numbers with AN to study this in ALSPAC.

## Conclusion

Disordered eating behaviours at age 16 are more common in all-girl schools, and in schools with lower academic results. This suggests that interventions to identify students with eating disorders should be implemented across these schools in particular, to help them support their students with disordered eating behaviours.

## Electronic supplementary material

Below is the link to the electronic supplementary material.

Supplementary material 1 (DOCX 78 kb)
