# Supplementary material for: Do disordered eating behaviours in girls vary by school characteristics? A UK cohort study
Source: Eur Child Adolesc Psychiatry. 2018 Mar 15;27(11):1473–81. doi: 10.1007/s00787-018-1133-0 (PMC6447693; doi:10.1007/s00787-018-1133-0)
Supplement: Supplementary file 1 — Supplementary material 1 (DOCX 78 kb) [file 787_2018_1133_MOESM1_ESM.docx]

Figure 1a & 1b: Flow charts showing derivation of the analytical samples for ages 14 & 16

Core sample: 14676 live births (7160 girls)

Missing data on School Attended at Key Stage 4: 1900 girls

6: 5365

Missing data on purging at age 16:2965 girls

Complete Data: 5260 girls

Singletons alive at 1 year: 6594 girls

Complete data: 2295 girls

Complete data: 2285 girls

Missing data on bingeing at age 16: 10 girls

Complete data: 2203 girls

Missing data on birth order: 66 girls

Complete data: 2154 girls

Missing data on maternal education:49 girls

Missing data on occupational class: 95 girls

Complete data: 2059 girls

Complete data: 2269 girls

Missing data on fasting at age 16: 16 girls

Complete data: 1769 girls

Missing data on maternal ED: 282 girls

Core sample: 14676 live births (7160 girls)

Missing data on School Attended at Key Stage 3: 1563 girls

6: 5365

Complete Data: 5031 girls

Singletons alive at 1 year: 6594 girls

Complete data: 2416 girls

Complete data: 2404 girls

Missing data on body dissatisfaction age 14: 2615 girls

Complete data: 2331 girls

Missing data on birth order: 73 girls

Complete data: 2269 girls

Missing data on maternal education: 62 girls

Missing data on occupational class: 113 girls

Complete data: 2156 girls

Missing data on weight/shape concern age 14: 12 girls

Complete data: 2156 girls

Complete data: 2146 girls

Missing data on maternal history of ED: 0 girls

Missing data on EPDS at 32 weeks pregnancy: 10 girls

Complete data: 1777 girls

Missing data on EPDS at 32 weeks pregnancy: 8 girls

**Table 1: Comparison of disordered eating behaviours between schools with different characteristics (expressed as odds ratios (ORs) or regression coefficients); unadjusted models**

|  | Age 14 | | | Age 16 | | | | | | |
| --- | --- | --- | --- | --- | --- | --- | --- | --- | --- | --- |
|  | Body Dissatisfaction score (regression coeff, 95% Confidence Interval) (N=2146) | Weight/Shape concern (OR; 95% Confidence Interval) (N=2146) | Parental report of fear of/avoidance of fat (score ≥3) (OR; 95% Confidence Interval) (N=1770) | Fasting (OR, 95% Confidence Interval) (N=1769) | Purging (OR, 95% Confidence Interval) (N=1769) | Binge eating (OR, 95% Confidence Interval) (N=1769) | Any compensatory behaviour (OR, 95% Confidence Interval) (N=1769) | Any compensatory behaviour (DSM-5 intensity) (OR; 95% Confidence Interval) (N=1769) | Parental report of fear of/avoidance of fat (score ≥3) (OR, Confidence Interval) (N=1441) |  |
| All girl vs mixed schools | -0.93 (-2.68, 0.83), p=0.30 | 0.70 (0.37, 1.34), p=0.29 | **1.73 (0.93, 3.21), p=0.08** | 1.05 (0.69, 1.59), p=0.82 | 1.28 (0.75, 2.19), p=0.36 | 1.11 (0.71, 1.74), p=0.65 | **1.41 (1.00, 1.97), p=0.05** | 1.17 (0.70, 1.94), p=0.55 | 1.13 (0.67, 1.90), p=0.65 |  |
| Top 25% vs bottom 75% KS point score total | **-1.02 (-1.86, -0.19), p=0.02**  **(N=1790)** | **0.68 (0.51, 0.91), p=0.01**  **(N=1790)** | 0.75 (0.52, 1.10), p=0.14  (N=1477) | **0.61 (0.45, 0.81), p=0.001 (N=1768)** | 0.78 (0.53, 1.16), p=0.22 **(N=1768)** | 0.96 (0.71, 1.29), p=0.77 **(N=1768)** | 0.90 (0.72, 1.12), p=0.33 **(N=1768)** | **0.55 (0.38, 0.82), p=0.003 (N=1768)** | 0.82 (0.57, 1.19), p=0.30 (N=1440) |  |
| School nurse vs no school nurse | -0.56 (-1.24, 0.13), p=0.11 | **0.73 (0.58, 0.91), p=0.007** | 0.82 (0.60, 1.10), p=0.19 | 0.92 (0.72, 1.17), p=0.51 | 1.07 (0.77, 1.49), p=0.68 | 0.92 (0.71, 1.21), p=0.57 | 1.11 (0.91, 1.35), p=0.32 | 0.97 (0.72, 1.32), p=0.87 | 0.87 (0.62, 1.21), p=0.40 |  |
| ≤5% vs >5% on free school meals | 0.26 (-0.42, 0.94), p=0.46 | 1.15 (0.92, 1.44), p=0.22 | 1.12 (0.84, 1.50), p=0.45 | 0.99 (0.78, 1.27), p=0.95 | 0.89 (0.63, 1.25), p=0.50 | 1.02 (0.78, 1.34), p=0.89 | 0.93 (0.76, 1.13), p=0.46 | 0.99 (0.72, 1.34), p=0.92 | 1.13 (0.81, 1.56), p=0.48 |  |

**Table 2: Comparison of disordered eating behaviours between schools with different characteristics (expressed as odds ratios (ORs) or regression coefficients); adjusted for individual characteristics***

|  | Age 14 | | | Age 16 | | | | | | |
| --- | --- | --- | --- | --- | --- | --- | --- | --- | --- | --- |
| School characteristics | Body Dissatisfaction score (regression coeff, 95% Confidence Interval) (N=2146) | Weight/Shape concern above cut off (OR, 95% Confidence Interval) (N=2146) | Parental report of fear of/avoidance of fat (OR; 95% Confidence Interval) (N=1770) | Fasting (OR, 95% Confidence Interval) (N=1769) | Purging (OR, 95% Confidence Interval) (N=1769) | Binge eating OR, 95% Confidence Interval) (N=1769) | Any compensatory behaviour (OR, 95% Confidence Interval) (N=1769) | Any compensatory behaviour (DSM-5 intensity) (OR, 95% Confidence Interval) (N=1769) | Parental report of fear of/avoidance of fat (score ≥3) (OR, 95% Confidence Interval) (N=1441) |  |
| All girls vs mixed schools | -0.73 (-2.49, 1.02), p=0.41 | 0.72 (0.38, 1.38), p=0.33 | **1.77 (0.94, 3.31), p=0.08** | 1.11 (0.73, 1.69), p=0.63 | 1.39 (0.81, 2.40), p=0.24 | 1.11 (0.70, 1.75), p=0.67 | **1.43 (1.01, 2.01), p=0.04** | 1.27 (0.76, 2.13), p=0.36 | 1.07 (0.63, 1.83), p=0.79 |  |
| Top 25% vs bottom 75% KS point score total | -0.61 (-1.51, 0.29), p=0.18  (N=1790) | 0.81 (0.59, 1.11), p=0.20 (N=1790) | 0.90 (0.60, 1.36), p=0.63 (N=1477) | **0.62 (0.46, 0.83), p=0.002**  **(N=1768)** | 0.77 (0.51, 1.15), p=0.20 **(N=1768)** | 0.96 (0.71, 1.31), p=0.79 **(N=1768)** | 0.89 (0.71, 1.12), p=0.32 **(N=1768)** | **0.60 (0.40, 0.89), p=0.01 (N=1768)** | 0.83 (0.57, 1.21), p=0.33 (N=1436) |  |
| School nurse vs no school nurse | -0.19 (-0.89, 0.50), p=0.58 | **0.79 (0.62, 0.99), p=0.05** | 0.88 (0.65, 1.20), p=0.43 | 0.93 (0.73, 1.19), p=0.57 | 1.07 (0.76, 1.49), p=0.70 | 0.93 (0.71, 1.22), p=0.60 | 1.10 (0.90, 1.35), p=0.34 | 0.99 (0.73, 1.35), p=0.96 | 0.88 (0.63, 1.23), p=0.46 |  |
| ≤5% vs >5% on free school meals | -0.13 (-0.83, 0.56), p=0.71 | 1.04 (0.83, 1.31), p=0.72 | 1.03 (0.76, 1.39), p=0.85 | 0.95 (0.73, 1.22), p=0.67 | 0.88 (0.62, 1.26), p=0.50 | 1.01 (0.76, 1.33), p=0.96 | 0.92 (0.75, 1.13), p=0.42 | 0.87 (0.63, 1.20), p=0.40 | 1.11 (0.79, 1.56), p=0.54 |  |

*birth order, maternal age, maternal education, parental social class, maternal depression, maternal eating disorder

**Table 3: Comparison of disordered eating behaviours between schools with different characteristics (expressed as odds ratios (ORs) or regression coefficients); adjusted for school characteristics***

|  | Age 14 | | | Age 16 | | | | | | |
| --- | --- | --- | --- | --- | --- | --- | --- | --- | --- | --- |
|  | Body Dissatisfaction score (regression coeff, 95% Confidence Interval) (N=1790) | Weight/Shape concern above cut off (OR, 95% Confidence Interval) (N=1790) | Parental report of fear of/avoidance of fat (score ≥3) (OR, 95% Confidence Interval) (N=1477) | Fasting (OR, 95% Confidence Interval) (N=1768) | Purging (OR, 95% Confidence Interval) (N=1768) | Binge eating (OR, 95% Confidence Interval) (N=1768) | Any compensatory behaviour (OR, 95% Confidence Interval) (N=1768) | Any compensatory behaviour (DSM-5 intensity) (OR, 95% Confidence Interval) (N=1768) | Parental report of fear of/avoidance of fat (score ≥3) (OR, 95%Confidence Interval) (N=1440) |  |
| All girls vs mixed schools | -0.87 (-2.93, 1.18), p=0.41 | 0.68 (0.31, 1.53), p=0.36 | **2.05 (0.99, 4.23), p=0.05** | 1.11 (0.72, 1.71), p=0.63 | 1.32 (0.76, 2.30), p=0.33 | 1.11 (0.70, 1.78), p=0.65 | **1.46 (1.03, 2.08), p=0.03** | 1.27 (0.75, 2.15), p=0.37 | 1.18 (0.68, 2.04), p=0.55 |  |
| Top 25% vs bottom 75% KS point score total | **-0.94 (-1.79, -0.09), p=0.03** | **0.71 (0.53, 0.96), p=0.03** | 0.75 (0.51, 1.09), p=0.13 | **0.60 (0.45, 0.81), p=0.001** | 0.77 (0.52, 1.14), p=0.19 | 0.95 (0.70, 1.28), p=0.73 | 0.88 (0.70, 1.09), p=0.24 | **0.55 (0.37, 0.81), p=0.003** | 0.81 (0.56, 1.18), p=0.27 |  |
| School nurse vs no school nurse | -0.41 (-1.19, 0.37), p=0.30 | 0.78 (0.60, 1.01), p=0.06 | 0.81 (0.57, 1.14), p=0.23 | 0.92 (0.72, 1.18), p=0.52 | 1.08 (0.77, 1.51), p=0.67 | 0.94 (0.71, 1.24), p=0.65 | 1.13 (0.92, 1.38), p=0.25 | 0.99 (0.72, 1.35), p=0.93 | 0.89 (0.64, 1.25), p=0.51 |  |
| ≤5% vs >5% on free school meals | 0.03 (-0.75, 0.80), p=0.95 | 1.04 (0.81, 1.33), p=0.77 | 1.06 (0.76, 1.49), p=0.72 | 0.99 (0.77, 1.28), p=0.96 | 0.93 (0.65, 1.33), p=0.71 | 1.01 (0.76, 1.34), p=0.95 | 0.99 (0.80, 1.22), p=0.93 | 1.01 (0.73, 1.40), p=0.94 | 1.13 (0.81, 1.59), p=0.48 |  |

*all girls vs mixed school, top 25% vs bottom 75% KS point score total, school nurse; ≤5% vs >5% on free school meals
